# The Role of Metformin Response in Lipid Metabolism in Patients with Recent-Onset Type 2 Diabetes: HbA1c Level as a Criterion for Designating Patients as Responders or Nonresponders to Metformin

**DOI:** 10.1371/journal.pone.0151543

**Published:** 2016-03-15

**Authors:** Zahra Kashi, Abdolkarim Mahrooz, Anvarsadat Kianmehr, Ahad Alizadeh

**Affiliations:** 1 Diabetes Research Center, Imam Teaching Hospital, Mazandaran University of Medical Sciences, Sari, Iran; 2 Immunogenetic Research Center, Mazandaran University of Medical Sciences, Sari, Iran; 3 Department of Clinical Biochemistry and Genetics, Faculty of Medicine, Mazandaran University of Medical Sciences, Sari, Iran; 4 Biochemistry and Metabolic Disorders Research Center, Golestan University of Medical Sciences, Gorgan, Iran; 5 Department of Medical Biotechnology, School of Advanced Technologies in Medicine, Golestan University of Medical Sciences, Gorgan, Iran; 6 Department of Epidemiology and Reproductive Health, Reproductive Epidemiology Research Center, Royan Institute for Reproductive Biomedicine, ACECR, Tehran, Iran; Northeast Ohio Medical University, UNITED STATES

## Abstract

**Background:**

In this study, we investigated whether response to metformin, the most frequently drug for diabetes treatment, influences the therapeutic effects of antilipidemic medication in newly diagnosed patients with type 2 diabetes mellitus (T2DM).

**Methods:**

A total of 150 patients with T2DM were classified into two groups following 3 months of metformin therapy (1000 mg twice daily): responders (patients showing ≥1% reduction in HbA1c from baseline) and nonresponders (patients showing <1% reduction in HbA1c from baseline). The patients received atorvastatin 20 mg, gemfibrozil 300 mg, or atorvastatin 20 mg and gemfibrozil 300 mg daily.

**Principal Findings:**

HbA1c and fasting glucose levels were significantly different between baseline and 3 months among responders receiving atorvastatin; however, these differences were not statistically significant in nonresponders. Atherogenic ratios of low-density lipoprotein cholesterol to high-density lipoprotein cholesterol (LDL-C/HDL-C; p = 0.002), total cholesterol to HDL-C (TC/HDL-C; p<0.001) and AIP (the atherogenic index of plasma; p = 0.004) decreased significantly in responders receiving atorvastatin than in nonresponders. Moreover, responders receiving atorvastatin showed a significant increase in HDL-C levels but nonresponders receiving atorvastatin did not (p = 0.007). The multivariate model identified a significant association between metformin response (as the independent variable) and TG, TC, HDL-C and LDL-C (dependent variables; Wilk's λ = 0.927, p = 0.036).

**Conclusions:**

Metformin response affects therapeutic outcomes of atorvastatin on atherogenic lipid markers in patients newly diagnosed with T2DM. Metformin has a greater impact on BMI in responders of metformin compared to nonresponders. Adoption of better therapeutic strategies for reducing atherogenic lipid markers may be necessary for metformin nonresponders.

## Introduction

Type 2 diabetes mellitus (T2DM) influences plasma lipids and lipoproteins. Patients with T2DM often develop dyslipidemia that increases risk of cardiovascular diseases (CVDs) [1,2]. Studies have shown that approximately 80% patients with T2DM die due to complications resulting from CVD [3]. Meta-analyses indicate that statins and fibrates decrease the risk of CVD [2]. These studies recommend that lipid-lowering therapies, particularly statin therapy, should be administered to prevent CVD mortality in diabetic patients [2].

The American Diabetes Association (ADA) and the European Association for the Study of Diabetes (EASD) recommend lifestyle modifications and initiation of metformin therapy in newly diagnosed patients with T2DM [4]. Metformin is the most frequently used antidiabetic drug for the management of T2DM [4,5]. It inhibits hepatic glucose production, increases glucose uptake, suppresses the gastric absorption of glucose, and serves as an insulin sensitizer [4,6,7]. Metformin has pleiotropic effects and can be useful in lipid metabolism, diabetic cardiomyopathy and vascular function [4]. Activation of AMP-activated protein kinase appears to be the main molecular mechanism of metformin action [4]. Variability in the response to metformin is one of the most important problems in the efficacy of the drug [8].

The importance of clinical response to metformin in newly diagnosed patients with T2DM has been shown recently [8]. In many patients with T2DM, metformin is prescribed concomitantly with antilipidemic agents particularly statins. In this observational study, we evaluated whether response to metformin influences the therapeutic effects of antilipidemic medication on lipid and lipoprotein metabolism in newly diagnosed patients with T2DM.

## Materials and Methods

### Patients and study design

Patients with a diagnosis of type 2 diabetes mellitus (mean age ± standard deviation = 52.7 ± 10.7 years) and treated with metformin were enrolled in this study. All patients were newly diagnosed according to the WHO criteria [9] and underwent a physical examination, and information about personal habits, medical history, demographic parameters and medication use was obtained by questionnaire. Criteria for exclusion in the study were type 1 diabetes mellitus, previous history of renal failure, autoimmune and liver diseases, chronic diseases, and pregnancy in women. The patients were followed for three months in this study. During this period, they took 1 g of metformin twice a day. None of the patients were taking antidiabetic medication prior to their diabetes diagnosis.

Some studies have reported that there is no accepted cut-off value for dividing metformin users into responders and nonresponders to the drug [10]. On the basis of clinical experience, some investigators have selected a 0.5% reduction in HbA1c levels as the cut-off value for designating patients as responders or nonresponders [10]. However, in a systematic review, Sherifali and colleagues reported that, after three months of metformin therapy (doses up to 1500 mg per day), HbA1c levels were reduced by approximately 1% compared with placebo [11]. The authors reported that this reduction continued to exist for at least 10 months after treatment began, and they found little evidence for further reduction at higher doses. In our study, duration of treatment and dose of metformin were similar to the study of Sherifali and colleagues [11]. Therefore, we selected ≥1% and <1% reduction in HbA1c levels as the criteria for classifying diabetic patients as metformin responders and nonresponders, respectively. Accordingly, patients were classified into two groups: (1) responders (n = 69) (patients showing ≥1% reduction in HbA1c levels from baseline) and (2) nonresponders (n = 81) (patients showing <1% reduction in HbA1c levels from baseline).

Out of 150 participants, 118 were women (of which 56 were responders and 62 were nonresponders) and 32 were men (of which 12 were responders and 20 were nonresponders). The patients were receiving lipid-lowering therapy with the following drugs: 20 mg/day atorvastatin (n = 109), 300 mg/day gemfibrozil (n = 24), or both 20 mg/day atorvastatin and 300 mg/day gemfibrozil (n = 17). Patients received a constant dosage regimen during the three-month study period. In addition, 35 patients in the nonresponder group and 30 patients in the responder group received antihypertensive medication including losartan, an angiotensin-converting enzyme (ACE) inhibitor or a beta blocker. Patients with a body mass index (BMI) of <30 kg/m^2^ were considered as non-obese, whereas those with BMI ≥30 were considered as obese [12]. The study protocol was approved by the ethics committee at Mazandaran University of Medical Sciences (in accordance with the principles of the Helsinki Declaration), and all participants provided written informed consent to participate in the study.

### Biochemical Tests

Standard enzymatic methods were applied to assay values of total cholesterol (TC), triglycerides (TGs), high-density lipoprotein-cholesterol (HDL-C), fasting blood glucose (FBG), alanine aminotransferase (ALT) and aspartate aminotransferase (AST) after an overnight fast. The assays were performed on the Prestige analyzer (Japan). We used the Friedewald formula to determine values of low-density lipoprotein-cholesterol (LDL-C) [13]. The atherogenic index of plasma (AIP) was calculated from the log (TG/HDL-C). The HbA1c levels were quantified by boronate affinity technique (Axis-Shield PoC AS, Oslo, Norway; accuracy, failure <5%). All of the parameters were measured before and after 3 months of metformin therapy.

### Statistical analyses

Normality of the distributions of variables was assessed with the Kolmogorov–Smirnov test. Differences between parametric variables were tested with paired t test or independent t test. Wilcoxon signed-rank test or Mann–Whitney U test were used to compare nonparametric variables. Planned comparisons were accomplished with a multivariate analysis of variance (MANOVA). *P*-values less than 0.05 were accepted as statistically significant. Statistical analyses were conducted using R (version 3.0.1) and SPSS (version 16.0) software.

## Results

### Overall changes in the study parameters after three months of metformin treatment

Table 1 shows the changes in study variables after 3 months of metformin treatment. Most of these parameters, except AST levels, were significantly decreased after 3 months of treatment. Compared with baseline, HDL-C levels were increased after the treatment (p = 0.101).

**Table 1:** Change in the study parameters from baseline to three months ofmetformin therapy.

Parameter	Baseline	After 3 months	p value
Systolic blood pressure (mmHg)	130.37**±**15.52	125.38**±**16.66	<0.001
Diastolic blood pressure (mmHg)	80.30**±**9.7	76.40**±**9.59	<0.001
BMI (kg/m^2^)	31.18**±**5.2	30.60**±**5.23	<0.001
Fasting glucose (mmol/L)	7.87**±**1.50	7.16**±**1.83	<0.001
HbA1c (%)	7.65**±**0.81	7.00**±**1.15	<0.001
ALT (μkat/L)	0.42**±**0.17	0.41**±**0.17	0.042
AST (μkat/L)	0.42**±**0.19	0.41**±**0.19	0.647
TG (mmol/L)	2.11**±**0.90	1.85**±**0.69	<0.001
TC (mmol/L)	4.90**±**1.05	4.54**±**0.85	0.001
HDL-C (mmol/L)	1.21**±**0.39	1.26**±**0.37	0.101
LDL-C (mmol/L)	2.70**±**0.89	2.34**±**0.70	<0.001
LDL-C/HDL-C	2.46**±**1.09	2.00**±**0.77	<0.001
TC/HDL-C	4.31**±**1.50	3.82**±**1.09	<0.001
AIP^a^	0.58**±**0.23	0.51**±**0.22	<0.001

Data are means **±** SD

^a^ Atherogenic index of plasma (the logarithmic transformation of TG/HDL-C)

### Changes in the study parameters in each treatment group after three months of metformin treatment

As shown in Table 2, TG (p = 0.001), TC (p<0.001), LDL-C (p<0.001), LDL-C/HDL-C (p<0.001), TC/HDL-C (p<0.001) and AIP (p = 0.001) decreased significantly after 3 months of atorvastatin therapy compared with baseline. TG (p = 0.003) and AIP (p = 0.007) decreased significantly in patients receiving gemfibrozil therapy. TC (p = 0.041), TC/HDL-C (p = 0.01) and AIP (p = 0.034) showed significant changes in patients receiving combination therapy of atorvastatin and gemfibrozil. As expected, HbA1c and fasting glucose levels decreased significantly in each treatment group after the 3 month treatment compared with baseline levels before treatment. In all the three groups, BMI, and systolic and diastolic blood pressure decreased significantly after 3 months of treatment. The changes of ALT and AST were not statistically significant in all the three groups after the treatment.

**Table 2:** Change in the study parameters from baseline to three months of metformin therapy in all the three treatment groups.

Parameter	Gemfibrosil (n = 24)			Atorvastatin (n = 109)			Combination (n = 17)		
	Baseline	After 3 months	p value	Baseline	After 3 months	p value	Baseline	After 3 months	p value
Systolic blood pressure (mmHg)	130.44±13.31	125.83±11.95	0.03	130.53±15.12	123.61±17.34	<0.001	132.03±12.01	125.00±12.25	0.012
Diastolic blood pressure (mmHg)	81.88±7.91	76.46±8.14	0.004	79.77±9.29	76.58±9.71	<0.001	81.67±6.99	75.67±8.21	0.021
BMI (kg/m^2^)	32.14±5.51	31.41±5.48	<0.001	31.47±5.40	30.97±5.44	<0.001	33.51±5.26	32.49±5.37	0.003
Fasting glucose (mmol/L)	8.17±1.24	7.34±1.72	0.008	7.80±1.43	7.24±1.90	0.001	8.03±1.35	6.73±1.39	<0.001
HbA1c (%)	7.83±0.97	7.18±1.08	0.009	7.65±0.78	7.05±1.16	<0.001	7.85±1.00	6.95±1.03	0.011
ALT (μkat/L)	0.44±0.23	0.37±0.16	0.217	0.42±0.16	0.40±0.17	0.298	0.42±0.14	0.35±0.13	0.088
AST (μkat/L)	0.48±0.29	0.39±0.17	0.144	0.42±0.18	0.41±0.19	0.479	0.49±0.28	0.40±0.15	0.131
TG (mmol/L)	2.89±0.99	2.17±0.65	0.003	2.07±0.87	1.83±0.71	0.001	2.60±0.91	2.06±0.75	0.085
TC (mmol/L)	4.87±0.97	4.44±0.88	0.087	4.96±1.09	4.49±0.84	<0.001	5.08±1.05	4.39±0.87	0.041
HDL- C (mmol/L)	1.10±0.47	1.15±0.24	0.673	1.21±0.38	1.25±0.35	0.187	1.02±0.28	1.17±0.20	0.121
LDL- C (mmol/L)	2.56±0.83	2.37±1.03	0.435	2.81±0.93	2.32±0.73	<0.001	2.87±0.83	2.42±1.17	0.203
LDL-C/HDL-C	2.67±1.37	2.18±1.07	0.159	2.58±1.20	1.98±0.78	<0.001	3.06±1.49	2.15±1.09	0.071
TC/HDL-C	4.75±2.23	3.96±1.21	0.151	4.44±1.59	3.76±1.06	<0.001	5.38±2.21	3.76±1.05	0.01
AIP ^a^	0.78±0.22	0.62±0.19	0.007	0.58±0.23	0.51±0.25	0.001	0.75±0.23	0.58±0.20	0.034

Data are means **±** SD

^a^ Atherogenic index of plasma (the logarithmic transformation of TG/HDL-C)

### Differences between responders and nonresponders at baseline

Comparison of the study parameters between responders and nonresponders at baseline are shown in Table 3. There were no statistically significant differences between responders and nonresponders before treatment began with respect to most of the study parameters, except HbA1c levels (p = 0.003).

**Table 3:** Comparison of the study parameters between responders and nonresponders at baseline.

Parameter	Non-responder	Responder	p value
Systolic blood pressure (mmHg)	133.85**±**15.13	128.75**±**15.93	0.095
Diastolic blood pressure (mmHg)	81.37**±**10.19	79.75**±**9.97	0.069
BMI (kg/m^2^)	30.91**±**5.33	31.4**±**5.07	0.46
Fasting glucose (mmol/L)	7.69**±**1.35	8.07**±**1.68	0.215
HbA1c (%)	7.45**±**0.74	7.86**±**0.82	0.003
ALT (μkat/L)	0.43**±**0.15	0.41**±**0.19	0.094
AST (μkat/L)	0.43**±**0.18	0.39**±**0.18	0.154
TG (mmol/L)	2.10**±**0.97	2.13**±**0.81	0.499
TC (mmol/L)	4.79**±**1.12	5.02**±**0.93	0.132
HDL-C (mmol/L)	1.24**±**0.39	1.17**±**0.38	0.297
LDL-C (mmol/L)	2.60**±**0.86	2.83**±**0.90	0.072
LDL-C/HDL-C	2.26**±**0.85	2.70**±**1.31	0.102
TC/HDL-C	4.09**±**1.09	4.60**±**1.86	0.118
AIP^a^	0.57**±**0.22	0.61**±**0.24	0.346

Data are means **±** SD

^a^ Atherogenic index of plasma (the logarithmic transformation of TG/HDL-C)

### Changes in BMI, lipid, and lipoprotein levels in response to metformin treatment in each treatment group after three months

After metformin treatment, responders receiving atorvastatin showed significantly improved lipid and lipoprotein levels; however, only LDL-C and LDL-C/HDL-C levels were significantly changed in nonresponders receiving atorvastatin (Table 4). Moreover, the BMI was significantly decreased in both responders (p<0.001) and nonresponders (p = 0.003) receiving atorvastatin.

**Table 4:** Change in the study parameters from baseline to three months of metformin therapy in responders and non-responders receiving atorvastatin.

Parameter	Non-responders receiving atorvastatin (n = 62)			Responders receiving atorvastatin (n = 47)		
	Baseline	After 3 months	p value	Baseline	After 3 months	p value
Systolic blood pressure (mmHg)	133.40±15.12	127.64±13.75	0.001	126.60±14.49	118.47±20.25	0.009
Diastolic blood pressure (mmHg)	80.98±9.82	77.41±10.55	0.004	78.19±8.50	75.64±8.57	0.058
BMI (kg/m^2^)	31.05±5.48	30.68±5.44	0.003	31.97±5.36	31.36±5.53	<0.001
Fasting glucose (mmol/L)	7.73±1.36	7.69±2.22	0.862	7.89±1.53	6.63±1.15	<0.001
HbA1c (%)	7.45±0.72	7.61±1.13	0.14	7.91±0.78	6.31±0.69	<0.001
ALT (μkat/L)	0.43±0.15	0.43±0.20	0.944	0.42±0.18	0.37±0.12	0.052
AST (μkat/L)	0.44±0.19	0.43±0.22	0.78	0.40±0.16	0.38±0.13	0.401
TG (mmol/L)	2.04±0.92	1.91±0.77	0.087	2.11±0.82	1.73±0.63	0.006
TC (mmol/L)	4.91±1.18	4.62±0.89	0.11	5.03±0.97	4.32±0.74	<0.001
HDL- C (mmol/L)	1.30±0.42	1.25±0.34	0.445	1.09±0.27	1.26±0.35	0.002
LDL- C (mmol/L)	2.71±0.93	2.40±0.75	0.037	2.95±0.91	2.22±0.69	<0.001
LDL-C/HDL-C	2.28±0.90	2.03±0.76	0.049	2.95±1.41	1.91±0.82	<0.001
TC/HDL-C	4.02±1.10	3.85±1.06	0.237	4.98±1.93	3.63±1.05	<0.001
AIP ^a^	0.54±0.22	0.52±0.23	0.353	0.63±0.25	0.48±0.21	<0.001

Data are means **±** SD

^a^ Atherogenic index of plasma (the logarithmic transformation of TG/HDL-C)

As indicated in Table 5, atherogenic ratios of LDL-C/HDL-C (p = 0.002), TC/HDL-C (p<0.001) and the AIP (p = 0.004) decreased significantly in responders receiving atorvastatin but did not decrease significantly in nonresponders. Moreover, responders receiving atorvastatin showed a significant increase in HDL-C levels but nonresponders receiving atorvastatin did not (p = 0.007). In addition, the reduction in TC (p = 0.066) and LDL-C (p = 0.063) levels reached borderline statistical significance in responders receiving atorvastatin. Such significant results were not observed for the gemfibrozil and combination groups. The decrease in BMI was higher in responders than in nonresponders across all three treatment arms; however, there were no significant differences between the three treatment groups.

**Table 5:** Differences between baseline parameter levels and levels after three months of treatment, according to metformin response and treatment group.

Parameter	Gemfibrozil			Atorvastatin			Combination		
	Non-responder (n = 14)	Responder (n = 10)	p	Non-responder (n = 62)	Responder (n = 47)	p	Non-responder (n = 8)	Responder (n = 9)	p
Δ (Systolic blood pressure) (mmHg)	2.89±10.13	7.00±9.19	0.32	5.76±13.23	8.13±20.35	0.467	6.50±9.39	7.50±10.00	0.846
Δ (Diastolic blood pressure) (mmHg)	5.71±7.30	5.00±9.72	0.839	3.57±9.38	2.55±9.02	0.570	7.14±6.99	5.00±10.69	0.659
Δ BMI (kg/m^2^)	0.55±1.03	1.01±.77	0.259	0.37±0.93	0.61±1.01	0.21	0.87±1.32	1.19±0.77	0.588
Δ (Fasting glucose) (mmol/L)	0.02±1.11	1.93±0.90	<0.001	0.03±1.79	1.29±1.46	<0.001	0.71±0.73	1.81±0.90	0.024
Δ HbA1c (%)	−0.09±0.64	1.68±0.74	<0.001	−0.16±0.83	1.60±0.56	<0.001	−0.09±0.64	1.76±0.82	<0.001
Δ ALT (μkat/L)	0.01±0.24	0.14±0.32	0.28	−0.02±0.21	0.04±0.16	0.195	0.05±0.04	0.07±0.18	0.797
Δ AST (μkat/L)	0.03±0.26	0.16±0.33	0.3	0.07±0.21	0.02±0.16	0.746	0.09±0.25	0.09±0.23	0.99
Δ TG (mmol/L)	0.76±0.82	0.64±1.34	0.785	0.17±0.76	0.37±0.88	0.207	0.58±0.65	0.50±1.47	0.905
Δ TC (mmol/L)	0.18±1.15	0.77±0.96	0.217	0.28±1.35	0.71±0.92	0.066	0.46±1.42	0.88±0.97	0.518
Δ HDL-C (mmol/L)	−0.11±0.32	0.04±0.72	0.469	0.04±0.40	−0.16±0.35	0.007	−0.04±0.27	−0.22±0.36	0.334
Δ LDL-C (mmol/L)	−0.01±1.26	0.50±1.24	0.336	0.31±1.13	0.72±1.06	0.063	0.08±1.75	0.81±1.11	0.357
Δ LDL-C/HDL-C	0.27±1.28	0.86±2.22	0.43	0.27±1.01	1.05±1.46	0.002	0.29±1.66	1.51±1.97	0.242
Δ TC/HDL-C	0.90±1.29	0.75±3.93	0.899	0.20±1.27	1.34±1.85	<0.001	1.19±1.20	2.15±2.66	0.433
Δ AIP^a^	0.16±0.18	0.15±0.34	0.886	0.02±0.19	0.15±0.25	0.004	0.11±0.16	0.22±0.34	0.493

Data are means **±** SD. Δ indicates the difference between baseline and after three months of metformin therapy (i.e., before minus after).

^a^ Atherogenic index of plasma (the logarithmic transformation of TG/HDL-C)

The multivariate model identified a significant association between metformin response (as the independent variable) and TG, TC, HDL-C and LDL-C (dependent variables; Wilk's λ = 0.927, p = 0.036). In these analyses, the results were adjusted for covariates such as age, gender, and BMI.

### Changes in HbA1c and fasting glucose levels in response to metformin treatment in each treatment group after three months

HbA1c and fasting glucose levels were significantly different between baseline and 3 months among responders receiving atorvastatin; however, these differences were not statistically significant in nonresponders (Table 4). In each of the three treatment groups, the reduction in HbA1c and fasting glucose levels was much higher (statistically significant) in responders than in nonresponders (Table 5).

## Discussion

Because of the importance of metformin response in the efficacy of the drug and the role of metformin in lipid metabolism, we tested the hypothesis that clinical response to metformin may influence the lipid-altering effects of atorvastatin, gemfibrozil, or atorvastatin and gemfibrozil combination therapy in patients with T2DM.

Our findings showed that atorvastatin and gemfibrozil combination therapy was no more effective in lowering the levels and ratios of atherogenic lipids than the atorvastatin monotherapy. In general, atorvastatin was superior in improving the lipid profile than gemfibrozil monotherapy or atorvastatin and gemfibrozil combination therapy. Contradictory reports are available on the different effects of statin monotherapy and statin and fibrate combination therapy on lipid parameters. Our results are consistent with the findings of a recent study by Rubenfire et al. [14], which reported that statin and fibrate combination therapy showed no efficacy in some trials. They stated that this combination therapy should be used as a secondary option because it may be associated with serious muscle toxicity. Furthermore, a study by Ginsberg and colleagues showed no differences between fenofibrate and simvastatin combination therapy and simvastatin monotherapy with respect to the reduction in the rates of cardiovascular events in high-risk patients with T2DM [15].

Before conducting metformin response analyses, baseline characteristics between responders and nonresponders were compared. Then, the covariates that were significantly different between responders and nonresponders were adjusted for further analyses.

When we analyzed the lipid and lipoprotein levels with respect to metformin response in each treatment group, our findings showed that all lipid and lipoprotein levels were significantly improved in responders receiving atorvastatin. These results were not observed with gemfibrozil monotherapy or combination therapy. These results indicate that metformin response influence atorvastatin therapy in newly diagnosed patients with T2DM. In previous studies, although metformin moderately improved the lipid profile, there were inconsistencies in its effects on lipid parameters [1]. The differing findings are partially related to the variability in response to the drug. To the best of our knowledge, there have been no previous studies that evaluated the effect of metformin response on the therapeutic outcomes of lipid-lowering agents in patients with T2DM.

Drug response and disposition could be affected by various factors such as genetics, organ function and nature of the disease [16,17]. Poor response to metformin may result from variability in metformin transporters, which play a role in the metabolism of the drug [17, 18]. For examole, polymorphisms, particularly those with reduced functions in the genes of organic cation transporters (OCTs) contribute to the variations in response to this important drug [18–20].

What factors contribute to the role of metformin response in the therapeutic effects of atorvastatin? One factor may be related to the potential role of oral antidiabetic drug (OAD) uptake transporters in hepatocytes [21, 22]. Uptake mechanisms can be important for the function of drugs such as statins, which are widely used concomitant with OADs in patients with T2DM [23]. Konig et al. stated that genetic variation in the genes encoding uptake transporters can contribute to interindividual variations in drug impact [23]. The uptake transporter organic anion transporting polypeptide 1B1 (OATP1B1) is able to transport a variety of endogenous substances, as well as drugs such as statins. This transporter is expressed at high levels in the liver, which is the predominant site of action of metformin[23]. Reports have shown that polymorphism in the gene for OATP1B1 can affect the AUC (area under the curve) for repaglinide, which is an antidiabetic drug [23]. Therefore, such uptake-transporter-mediated drug interactions may be involved in the differential effects of atorvastatin in metformin responders and nonresponders. However, detailed in vivo studies are needed to clarify the drug interactions.

In this study, BMI decreased significantly in each treatment group. When analyses were performed with respect to metformin response, our results showed that the BMI decrease was higher in responders than in nonresponders in all the three treatment groups. In other words, metformin has a greater effect on BMI in responders than in nonresponders.

Results of several studies indicate that TC/HDL-C and LDL-C/HDL-C ratios could be better predictors of patients at high risk for atherosclerosis and CVD than single lipid parameters [24]. Our findings showed that these atherogenic ratios were reduced significantly only in responders receiving atorvastatin and not in nonresponders. Therefore, atorvastatin is more effective in decreasing the risk of CVDs in patients with T2DM who respond to metformin than those who do not respond to metformin. In the other words, CVD risk is higher in diabetic patients who do not respond to metformin and who concomitantly receive atorvastatin than in patients who respond to metformin and who concomitantly receive atorvastatin. AIP (logarithmically transformed ratio of TG/HDL-C) significantly decreased in all three treatment groups. However, when analyses were performed with respect to metformin response, our results showed that the atherogenic index decreased significantly only in responders receiving atorvastatin and not in nonresponders. Because AIP is inversely correlated with the size of LDL particles, lower AIP indicates larger LDL particles, which in turn would imply a decreased risk of atherosclerosis [25,26]. In the other words, atorvastatin is more effective in decreasing this atherogenic index in responders of metformin than in nonresponders; this in turn indicates higher atherogenicity in nonresponders. Therefore, patients with T2DM who do not respond to metformin may need better treatment strategies for lowering atherogenic lipids. According to an algorithm proposed in a review article [14], niacin or high dose omega-3 fatty acid therapy could be used in patients with an atherogenic lipid phenotype, secondary to lifestyle modifications and the use of a potent statin such as atorvastatin as the first treatment priority. It appears that this algorithm may be particularly effective for metformin nonresponders receiving atorvastatin. However, further clinical studies are needed to better understand the effects of these therapies.

It should be noted that we also analyzed the difference between the values for atherogenic lipids and ratios at baseline and after three months according to metformin response in individuals who were not receiving any antilipidemic medication. In this analysis, there were no significant differences between responders and nonresponders with respect to the parameters of this study (results not shown).

In the present study, within three months of treatment, diet and physical activity is recommended for all patients equally. However, the factors may be considered as the limitation of this study.

In summary, we have shown that the clinical response to metformin, in addition to lowering BMI and glucose levels, influence the therapeutic outcomes of the lipid-lowering drug atorvastatin in patients newly diagnosed with T2DM. Uptake-transporter-mediated drug interactions in the liver may potentially be involved in the differential action of atorvastatin in metformin responders and nonresponders. According to our findings, atorvastatin is more effective in reducing the atherogenic lipid parameters [TC/HDL, LDL/HDL and log (TG/HDL)] in metformin responders than in nonresponders. CVD risk may therefore be higher in metformin nonresponders who receive atrovastatin than in metformin responders who receive this lipid-lowering drug. As a result, adoption of better therapeutic strategies for reducing atherogenic lipids may be necessary for metformin nonresponders. Further investigations with a longer treatment period and larger sample size should be performed to validate whether metformin response influences the therapeutic effects of lipid-altering medication in patients with T2DM.
